# Robotic bilateral axillo-breast versus endoscopic bilateral areola thyroidectomy outcomes of 757 patients

**DOI:** 10.3389/fendo.2022.1029845

**Published:** 2023-01-20

**Authors:** Daqi Zhang, Cheng Wang, Chengqiu Sui, Kunlin Li, Mingyu Yang, Gaofeng Xue, Gianlorenzo Dionigi, Hoon Yub Kim, Hui Sun

**Affiliations:** ^1^ Division of Thyroid Surgery, Jilin Provincial Key Laboratory of Surgical Translational Medicine, China-Japan Union Hospital of Jilin University, Changchun, China; ^2^ Jilin Provincial Precision Medicine Laboratory of Molecular Biology and Translational Medicine on Differentiated Thyroid Carcinoma, China-Japan Union Hospital of Jilin University, Changchun, China; ^3^ Department of Pathophysiology and Transplantation, University of Milan, Milan, Italy; ^4^ Division of Surgery, Istituto Auxologico Italiano Instituto di Ricovero e Cura a Carattere Scientifico (IRCCS), Milan, Italy; ^5^ Korea University College of Medicine (KUMC) Thyroid Center, Department of Surgery, Korea University Hospital, Korea University College of Medicine, Seoul, Republic of Korea

**Keywords:** endoscopic surgery, thyroidectomy, robotic-assisted surgery, morbidity, cost, learning curve

## Abstract

**Background:**

Remote endoscopic thyroidectomy (ET) and robotic thyroidectomy (RT) seem to be beneficial in selected situations to avoid anterior neck scars. There are limited data in the literature to determine whether RT *via* a bilateral axillo-breast approach (RT-BABA) is superior to ET *via* a bilateral areolar approach (ET-BAA). Therefore, the aim of this study was to evaluate the surgical outcomes of RT-BABA versus ET-BAA.

**Methods:**

Between May 2013 and May 2022, 757 patients who underwent RT-BABA or ET-BAA at a high-volume Chinese thyroid center were included. Intraoperative and postoperative outcome parameters were collected and retrospectively analyzed. The moving average method was used to evaluate the learning curve.

**Results:**

The proportion of patients older than 45 years was greater in the RT group than in the ET group (14.8% vs. 7.4%, p < 0.001). The percentage of overweight patients was greater in the RT group (28.8% vs. 9.5%, p < 0.001). The number of patients treated for malignant lesions was higher in the RT group (86.8% vs. 75%, p < 0.001). The rate of thyroiditis was higher in the RT group (10.9% vs. 6.6%, p < 0.001). Surgical time was significantly shorter in the RT group (140 vs. 165min, p < 0.001). Drainage volume was higher in the RT group (100 vs. 85ml, p < 0.001). Postoperative hospital stay was shorter in the RT group (3.04 ± 0.44 vs. 3.67 ± 0.89 days, p < 0.001). The cost in the RT group was higher (49627 ± 2795 vs. 25094 ± 3368 yuan, p < 0.001). Transient vocal cord dysfunction was lower in the RT group (2.9% vs. 8.0%, p = 0.003). There was no significant difference between the two groups in the number of central lymph nodes sampled, positive lymph nodes, neural monitoring (EMG) results, and rate of transient hypoparathyroidism. The learning curve for RT was 26 cases, and the operative time for ET was constant throughout the study.

**Conclusions:**

RT-BABA is as safe and feasible as ET-BAA. RT-BABA performed better in some surgical outcomes. Further prospective studies are needed to confirm the safety of RT-BABA.

## Introduction

In recent decades, minimally invasive thyroid surgery has gained increasing popularity (1, 2). with the introduction of endoscopic techniques, several advantages have been demonstrated in terms of cosmesis, length of hospital stay, degree of postoperative pain, blood loss, voice quality, and faster return to normal activities (3–6). These benefits have also been demonstrated for oncologic resections (7).

The introduction of robotic-assisted systems took minimally invasive thyroid surgery to the next level. The robotic surgical system was introduced to thyroid surgery by Kang in 2009 (8). The Endowrist™ function with its improvement of comfort and maneuverability, filtering of tremor and movements by the computer system, high-resolution 3-dimensional binocular vision with the possibility of magnification, fluorescence, and finally a stable platform with a surgeon-operated camera can even be considered as a revolution and new dimension in thyroid surgery (2, 3, 9, 10). As a result, various endoscopic thyroidectomy approaches (ET), including transaxillary, bilateral areola, facelift, and transoral approaches, have been transferred to robotics (2).To our knowledge, few studies have compared surgical outcomes between robotic thyroidectomy *via* the bilateral axillary thoracic approach (RT-BABA) and endoscopic thyroidectomy *via* the bilateral areola approach (ET-BAA). Therefore, the aim of this study was to evaluate the surgical outcomes of RT-BABA versus ET-BAA.

## Materials and methods

### Study design

A retrospective, comparative observational study.

### Setting

A major thyroid surgery center, i.e., the Department of Thyroid Surgery, China-Japan Union Hospital of Jilin University, Changchun, China.

### Time frame

May 2013 to May 2022.

### Preoperative assessment

All patients were examined according to International guidelines (11). All surgical indications were approved by a multidisciplinary board.

### Inclusion and exclusion criteria

We retrospectively studied patients > 18 years old who underwent endoscopic thyroidectomy *via* a bilateral areola approach and robotic thyroidectomy *via* a bilateral axillo-breast approach. The detailed inclusion and exclusion criteria for ET and RT are summarized in **Table 1**. Only one surgeon (DQZ) had experience in robotic and endoscopic surgery. ET vs. RT were selected based on instrument availability and surgeon preference, with no specific selection criteria.

**Table 1:** Detailed inclusion and exclusion criteria for ET and RT.

Selection and exclusion criteria
Selection criteria
Papillary thyroid cancer with low-risk factors ^a^
Dominant benign nodule with a diameter <5 cm, whereas cystic nodule could be 6 cm or greater
The patients needed a cosmetic requirement
Exclusion criteria
General factors
Clinical history of radiation or surgery on the neck or chest
Preoperative dysfunction of voice cord
Thyroid-related factors
Advanced cancer
Local invasion
Posteriorly located lesions
Diffuse or adhesion or fixation enlargement of lymph node
Evidence available of local or distant metastases
Graves’ disease
Severe thyroiditis
Associated parathyroid disease

a Low risk factors including lesion size <4 cm, age <55 years, no prior radiation, no distant metastases, no lymph node metastases, no extrathyroidal extension, no aggressive variant, and no first-degree family history of thyroid carcinoma.

### Data collection

Data were obtained retrospectively from electronic databases. Demographic data (age, sex, and BMI) and outcome parameters were extracted, including conversion to open surgery, intraoperative and postoperative complications (30-day morbidity), operative time, postoperative length of hospital stay, blood loss, discharge home, costs, electromyographic (EMG) changes in recurrent laryngeal nerve (RLN) (12). The time from skin incision to closure was the operative time. Learning curve of two techniques had been made with moving average method, every 5 patients was a group (the moving average period was set to 5). The cost included the expense of perioperative examination and surgical treatment. Reason of RLN injury was repeatedly confirmed by viewing the video. All patients underwent fiberoptic laryngoscopy before surgery and on the first day after surgery (13). According to the examination results, normal vocal cord movement or vocal cord paralysis was detected (13). Those who recovered within 6 months were considered as temporary vocal cord paralysis, and those who did not recover within 6 months were considered as permanent (13). Hypoparathyroidism is defined as parathyroid hormone below 15pg/ml. Transient hypoparathyroidism recovering within 6 months, and permanent hypoparathyroidism lasting longer than 6 months (14). Discharge criteria consisted in adequate pain control with oral medication, and soft food well-tolerated, mobilization, and self-support.

### Technology overview and surgical techniques

**Figure 1:**
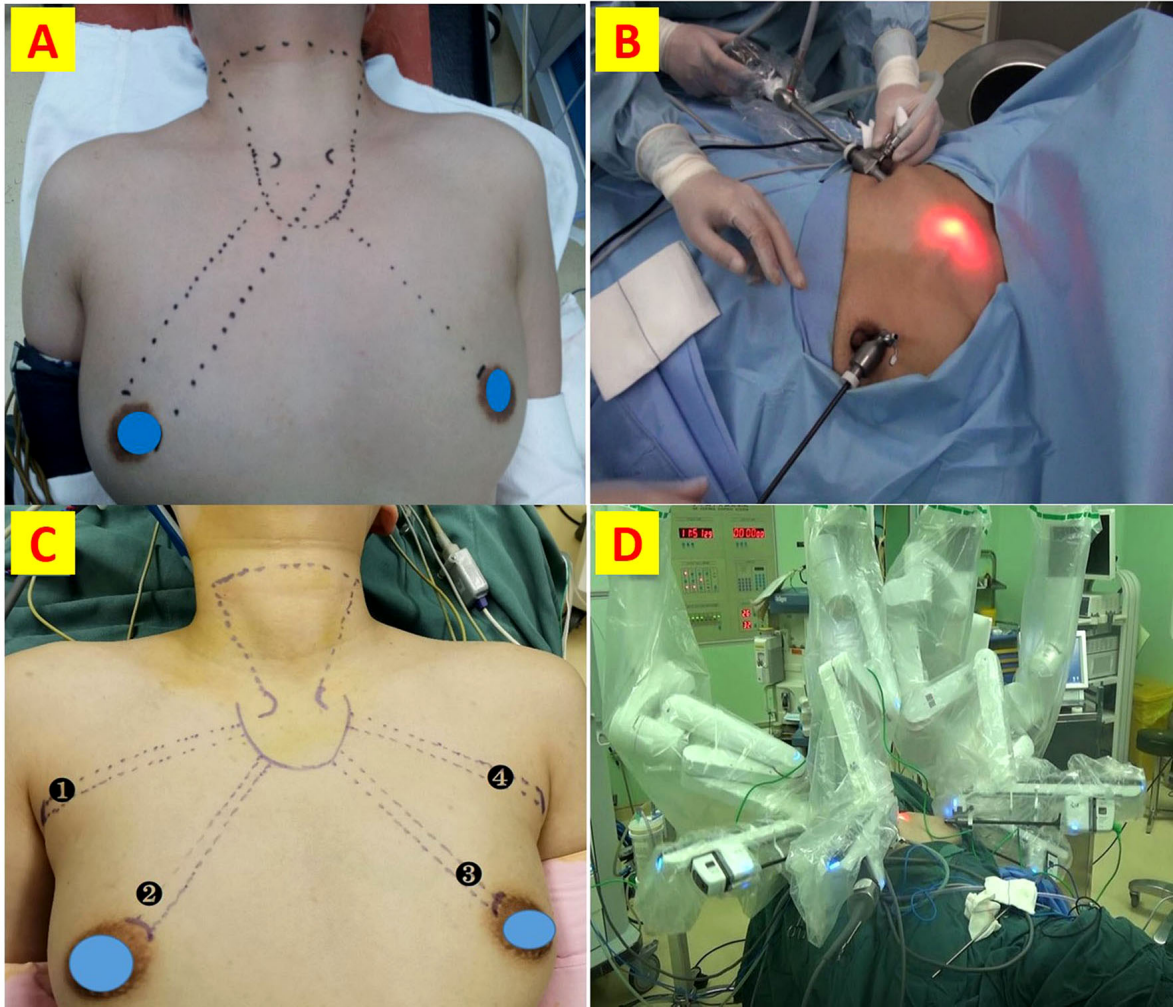
Detailed procedures for ET-BAA and RT-BABA. **(A)** Surgical incision and approach for ET; **(B)** port insertion for the bilateral areola approach; **(C)** Surgical incision and approach for RT; **(D)** robotic arm placement and surgical scenarios.

Detailed procedures for ET-BAA and RT-BABA have been reported previously (**Figure 1**) (3, 5, 14). ET-BAA was performed with Olympus endoscopic surgical system, and RT-BABA was performed with a fourth-generation da Vinci^®^ Surgical System (Intuitive Surgical, Sunnyvale, CA, USA) in this study. The main difference between two techniques was summarized in **Table 2**. ET-BAA consisted of a 10-mm curved incision made along the edge of the right areola at 2-4 of the clock to use a 10-mm trocar and a 30-degree endoscope. Bilateral 5-mm incisions were made along the borders of the areola, and two 5-mm trocars were used as main and auxiliary operating ports. CO2 was maintained at 6 mmHg. RT-BABA included two 10-mm axillary incisions and two 10-mm areolar incisions on the left and right sides. The right areolar incision was used as a camera port. The left areolar incision and bilateral axillary incision were each placed in the trocar for connection of the ultrasound scalpel, Maryland forceps, and grasping forceps. CO2 was continuously introduced at a pressure of 6 mmHg to maintain the surgical space.

**Table 2:** The main differences between the two technologies.

	RT	ET
Vision		
Enlarge	10-15 times	4-6 times
3D	yes	no
Stable	yes	no
Instrument		
slender	yes	yes
Rotate 360 degrees	yes	no
**Manipulator**	3	2

### Statistical analysis

Results were analyzed using SPSS Statistics version 23 (SPSS Inc., Chicago, IL, USA). Continuous variables are presented as means with standard deviations (mean ± SD) when the data were normally distributed and as medians with interquartile ranges (IQRs) when the data were abnormally distributed. Categorical variables are expressed as percentages. The independent samples t-test was used to compare continuous variables between the two groups. The Mann-Whitney test was used to compare discontinuous variables, whereas the chi-square test was used to compare categorical variables. Differences were considered statistically significant if the p-value was less than 0.05.

## Results

**Figure 2:**
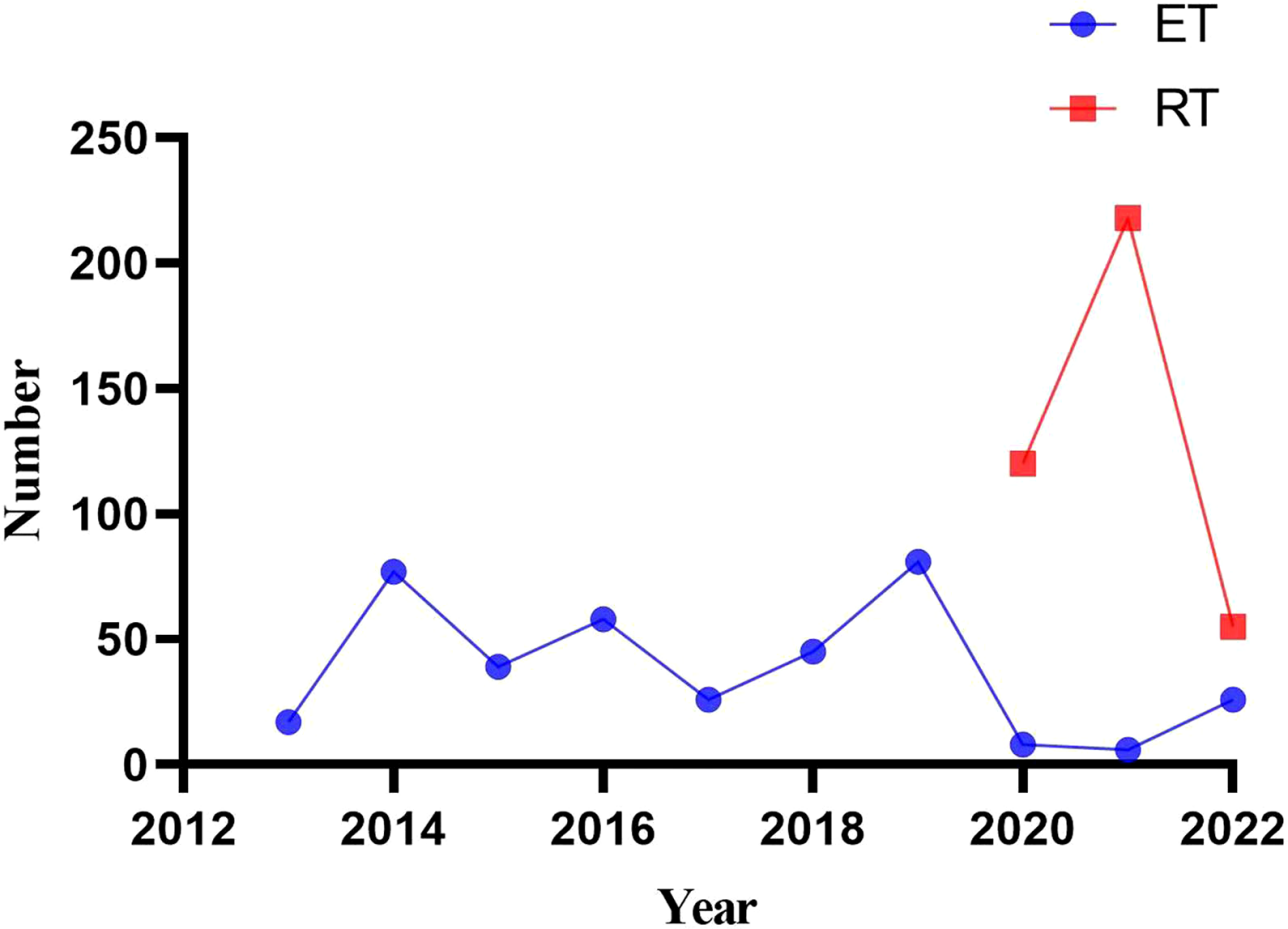
The changing trend of robotic and endoscopic thyroidectomy in our center from 2013 to 2022.

The detailed data of the patients are summarized in **Table 3**. A total of 757 patients were enrolled in this study (364 patients in the ET and 393 patients in the RT group). All surgeries were successfully completed, and there were no cases of conversion to open surgery. **Figure 2** reflects the changing trend of robotic and endoscopic thyroidectomy in our center from 2013 to 2022.

**Table 3:** Demographics, pathological characteristics and Perioperative outcomes of patients.

Characteristics	ET(*n*=364)	RT(*n*=393)	*P*-value
Age (median [IQR]), years	31(25, 38)	33(28, 40)	<0.001
<45	337(92.6)	335(85.2)	<0.001
≥45	27(7.4)	58(14.8)	
Sex			
Male	34(9.5)	38(9.7)	0.926
Female	325(90.5)	355(90.3)	
BMI(median [IQR]) ,kg/m^2^	22.5(19.3, 23.2)	21.2(20.5, 25.4)	<0.001
<25	319 (90.5)	280 (71.2)	<0.001
≥25	44 (9.5)	113 (28.8)	
Pathology			
Benign	91(25)	52 (13.2)	<0.001
Malignant	273(75)	341 (86.8)	
Tumor diameter, mm	5.8 ± 4.9	6.3 ± 4.4	0.135
Thyroiditis	24 (6.6)	43 (10.9)	0.035
LN status of CND			
Retrieved LNs	4.0 ± 3.1	4.4 ± 3.4	0.127
Positive LNs	0.8 ± 1.5	0.9 ± 1.5	0.558
Operation time(median [IQR]) ,min	165 (135, 200)	140 (120, 165)	<0.001
Total amount of drainage(median [IQR]) ,ml	85 (70, 100)	100 (85, 110)	<0.001
Postoperative hospital stay time(mean ± SD) ,day	3.67 ± 0.89	3.04 ± 0.44	<0.001
Cost(mean ± SD), yuan	25094 ± 3368	49627 ± 2795	<0.001
EMG changes of RLN	46 (12.6)	51 (12.9)	0.139
Reason of RLN injury			0.268
Compression	36 (78.3)	46 (90.2)	
Tumor invasion	2(4.3)	1 (2.0)	
Vascular heat conduction	8(17.4)	4 (7.8)	
Postoperative complication			
Transient VCD	29 (8.0)	11 (2.9)	<0.001
Permanent VCD	0	0	
Transient HPT	5(1.4)	1(0.3)	0.083
Permanent HPT	0	0	

VCD, Vocal cord dysfunction; HPT, Hypoparathyroidism.

### Demographic and pathologic characteristics

**Figure 3:**
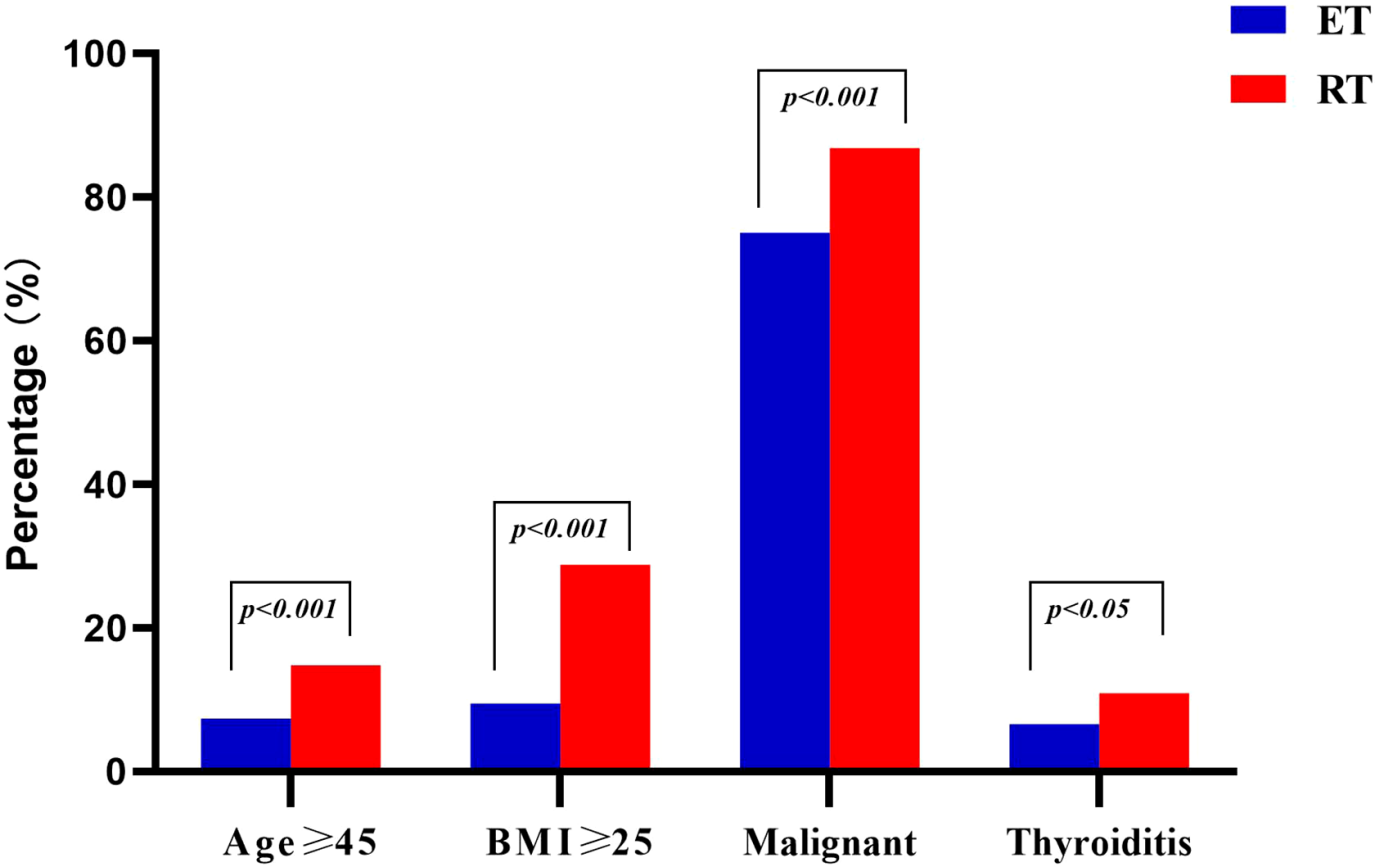
Comparison of demographics and pathological characteristics between ET and RT.

As shown in **Table 3**, patients in the RT group were significantly older (33 vs. 31 years, p < 0.001). In the RT group, 58 (14.8%) patients were older than 45 years and in the ET group, 27 (7.4%) were older (**Figure 3**). The median body mass index (BMI) was 22.5 kg/m^2^ in the RT group and 21.2 kg/m^2^ in the ET group (p < 0.001). The proportion of overweight patients (BMI ≥ 25 kg/m2) was greater in the RT group than in the ET group (p < 0.001) (**Figure 3**). The sex ratio was similar between the two groups (p > 0.05). Regarding the pathological results, there were 341 (86.8%) malignant lesions in the RT group and 273 (75%) in the ET group (**Figure 3**). Thyroiditis was detected in 43 patients in the RT group (10.9%) and 24 patients in the ET group (6.6%) (**Figure 3**). There was no significant difference in the number of central lymph nodes and positive lymph nodes sampled between the two groups (p > 0.05).

### Perioperative outcomes

**Figure 4:**
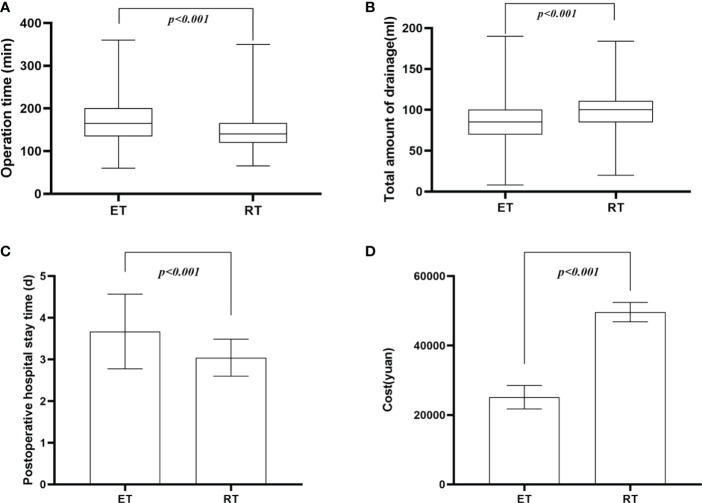
Comparative analysis for Perioperative outcomes . **(A)** operative time; **(B)** total amount of drainage; **(C)** postoperative hospital stay; **(D)** cost.

**Figure 5:**
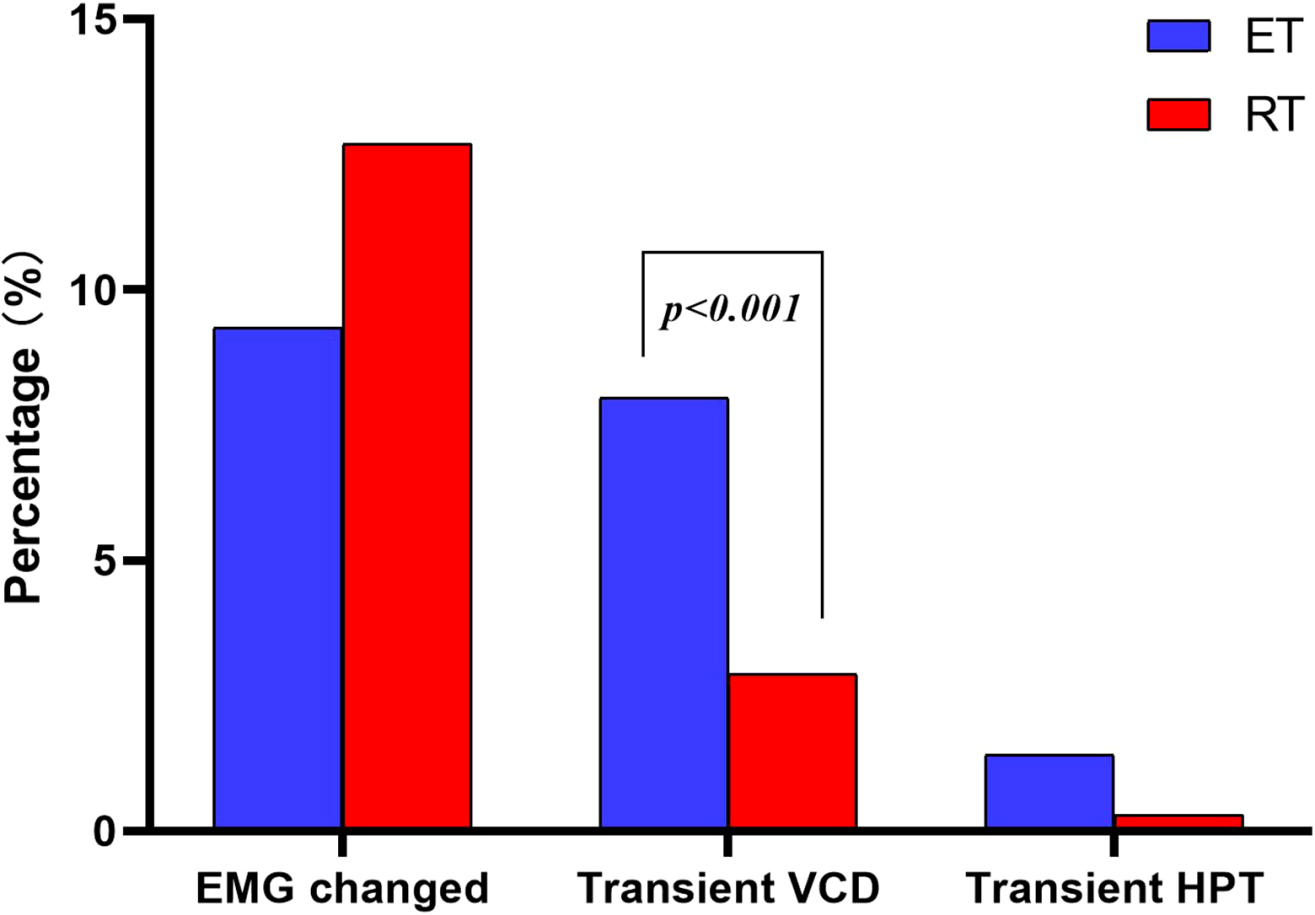
Comparison of postoperative complication between ET and RT.

The median operation time was significantly shorter in the RT group than in the ET group (140 vs. 165 min, p < 0.001) (**Figure 4A**). Total drainage volume was higher in the RT group than in the ET group (100 vs. 85 ml, p < 0.001) (**Figure 4B**). Postoperative hospital length of stay was shorter after robotic thyroidectomy (3.04 ± 0.44 vs. 3.67 ± 0.89 days, p < 0.001) (**Figure 4C**). Although there was no difference in intraoperative EMG changes of the RLN between the two groups (12.3% vs. 9.7%, p = 0.139), the rate of transient vocal cord dysfunction was lower in the RT group than in the ET group (2.9% vs. 8.0%, p = 0.003) (**Figure 5**). The elucidated mechanisms among 97 RLN lesions were included compression, vascular heat conduction and tumor invasion. In ET group, 36 cases (78.3%) were compression, 8 (17.4%) vascular heat conduction, 2 (4.3%)tumor invasion. In RT group, the main causes of RLN injury were compression (n=46, 90.2%), followed by vascular heat conduction, (n= 4,7.8%) and tumor invasion (n = 1, 2.0%). There was no difference in the reason of RLN injury between the two groups (p > 0.05).The rate of transient hypoparathyroidism was also similar in the two groups (**Figure 5**). The mean cost of robotic thyroidectomy was 49627 ± 2795 yuan compared with 25094 ± 3368 yuan for endoscopic thyroidectomy (**Figure 4D**) (P < 0.001).

### Learning curve

**Figure 6:**
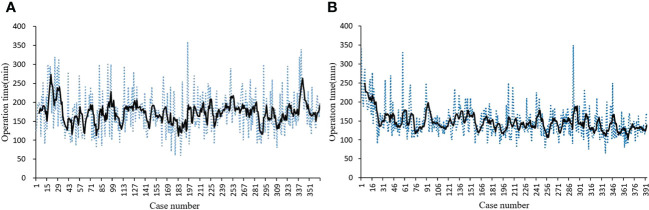
Learning curve. **(A)** Learning curve for ET-BAA; **(B)** Learning curve for RT-BABA.

In 396 consecutive robotic-assisted procedures, the learning curve was generated using the moving average method. The operation time decreased significantly after approximately 26 cases and gradually stabilized (**Figure 6A**). The operation time for ET was constant throughout the study (**Figure 6B**).

## Discussion

In recent decades, minimally invasive techniques have gained considerable importance in thyroid surgery (3–6). According to the latest studies, ET consolidates its advantages compared to the traditional open approach (6). Recently, authors have advocated the safety and satisfactory results of robotic thyroidectomy (3–6). As the **Table 2** shown, robotic surgical system has technical advantage compared with endoscopy. The da Vinci robotic surgical system provide a three-dimensional 15-fold magnified stable view, which eliminates the fatigue caused by the shaking of surgical field. Unlike ET, surgical instruments in RT provide surgeons with a wider range of stimulated movements than the human hand to complete flexible and natural operations, allowing for delicate operations in tight spaces. Furthermore, the appearance of the third robotic arm leads to a huge physical and labor saving compare to ET. However, the actual benefit of robotic thyroid surgery versus ET in the treatment of thyroid disease is still debated today. Preliminary results are encouraging and supportive of the robotic platform, but issues with operative time and cost present a significant obstacle to ultimately overcoming the robotic system versus ET.

In our study, we report several years of experience with mini-invasive surgery for thyroid disease at our institution involving a specialized endocrine surgeon. ET was performed *via* a bilateral areolar approach, and RT was performed *via* a bilateral axillo-breast approach. The previous studies in our center have shown that both approaches have favorable cosmetic effect (3, 15).

In our study, patients in RT group were older than those in ET group. On the one hand, it reflects the increasing acceptance of minimally invasive thyroid surgery, especially robotic thyroid surgery. On the other hand, minimally invasive thyroid surgery is no longer the patent of young women, and has also become the choice of many middle-aged and elderly patients.

We found RT group had 113 operations (28.8%) on overweight patients (BMI>25kg/m^2^), and ET group had 44(9.5%).This was mainly attribute to the technical superiority of robotic surgical system. A study performed in the Korea reported that BMI was an independent risk factor for prolonged operative time and that the longer operative time was due to the extended times needed for flap dissection (16). But in a retrospective study comparing patients who had undergone the RT-BABA, there was no difference in operative time between patients with BMI below 25 kg/m^2^ and those above 25kg/m^2^ (17). Regarding pathological results, RT group had more patients with malignant tumor, and patients with thyroiditis. Although there was no statistic difference in the ability of lymph node dissection, the RT group showed a increasing trend in the number of central lymph nodes and positive lymph nodes.

Presently, RT-BABA appears to require a shorter operation time compared with ET-BAA. Similar to other literatures (18, 19), there is an obvious leaning curve for RT. The learning curve of our study is 26 cases. However, no obvious learning curve was observed in ET. The total amount of drainage was greater in RT group than because we adopted a strategy of increasing flushing in RT. Although the findings revealed that ET group had similar risk of RLN injury, the incidence of temporary postoperative vocal cord palsy was significantly higher in ET group (8.0%) compared to RT group (2.9%).

The strength of this study is the large sample size. The study was conducted in a center with high volume and established experience in minimally invasive surgery. However, this study has some limitations. It is a monocentric study of a nonrandomized patient series based on a retrospective database. Further multicenter prospective randomized studies are needed to confirm our results for a larger number of surgeons in comparable high-volume centers.

In conclusion, the real superiority of RT is seen in more difficult cases, including elderly patients, overweight patients, bilateral thyroidectomy and thyroiditis. In addition, thyroidectomies seem to receive greater benefits from the robotic approach, with a lower complication rate (RLN injury), operative time, hospital stay, learning curve than ET. However, robotic thyroidectomy also has some drawbacks. First, RT is more expensive than ET. Secondly, RT lacks haptic feedback, which places higher demands on the operator’s eye-hand coordination. Furthermore, real-time suction cannot be performed unless occupying one robotic arm. It is believed that with the upgrading of technology and the innovation of theory, robotic thyroidectomy will be further promoted.

## Data availability statement

The raw data supporting the conclusions of this article will be made available by the authors, without undue reservation.

## Ethics statement

The studies involving human participants were reviewed and approved by This study was approved by the Medical Ethics Committee (protocol no.2019002). The patients/participants provided their written informed consent to participate in this study.

## Author contributions

All authors listed have made a substantial, direct, and intellectual contribution to the work and approved it for publication.
